# Tuning the Reactivity of Perfluoropolyether-Functionalized Aluminum Nanoparticles by the Reaction Interface Fuel-Oxidizer Ratio

**DOI:** 10.3390/nano12030530

**Published:** 2022-02-03

**Authors:** Chengcheng Wu, Jianxin Nie, Shengwei Li, Wei Wang, Qi Pan, Xueyong Guo

**Affiliations:** 1 State Key Laboratory of Explosion Science and Technology, Beijing Institute of Technology, Beijing 100081, China; 3120185179@bit.edu.cn (C.W.); niejx@bit.edu.cn (J.N.); 3120210240@bit.edu.cn (S.L.); 3220185029@bit.edu.cn (W.W.); panqi97@163.com (Q.P.); 2 Beijing Institute of Space Long March Vehicle, China Academy of Launch Vehicle Technology, Beijing 100074, China

**Keywords:** Al NPs, perfluoropolyether-functionalized, oxidation mechanism, reaction interface Fuel-Oxidizer ratio

## Abstract

To deepen the oxidation depth and promote the exothermic reaction of aluminum nanoparticles (Al NPs), this work constructed perfluoropolyether-functionalized Al NPs by using a facile fabrication method. It was determined that perfluoropolyether (PFPE) was uniformly distributed on the surface of the Al NPs with no obvious agglomeration by micro-structure analysis. Thermogravimetric analysis (TGA), differential scanning calorimetry (DSC), microcomputer automatic calorimeter (MAC), and combustion and ignition experiments were performed for varying percentages of PFPE blended with Al NPs to examine the reaction kinetics and combustion performance. It was revealed that the oxidation mechanism of PFPE-functionalized Al NPs at a slow heating rate was regulated by the reaction interface Fuel-Oxidizer ratio. Due to the enlarged Fuel-Oxidizer contact surface area, fluorine atoms could adequately decompose the inert alumina shell surrounding the Al NPs, optimizing the combustion process of Al NPs. The analytical X-ray diffraction (XRD) pattern results confirmed the existence of aluminum trifluoride in combustion products, providing insights into the oxidation mechanism of Al NPs. The obtained results indicated that PFPE participated in the oxidation of Al NPs and improved the overall reactivity of Al NPs.

## 1. Introduction

Aluminum (Al) has been considered as a reactive metal with potentially superior exothermic performance (83.8 kJ·cm^−3^/31.05 kJ·g^−1^) [1] and has been widely used in the fields of propellants, explosives, and pyrotechnics [2,3,4]. However, its application has often been restrained due to the native dense oxide layer (Al_2_O_3_) passivating on the surface, hindering the diffusion of oxygen throughout Al particles in the combustion process [5,6,7]. Meanwhile, the combustion process of Al particles usually accompanies ignition delay, incomplete combustion, and so on [8,9,10]. Moreover, the larger the size of Al particles is, the longer the path of heat conduction will be [11]. This phenomenon is a minor point, but it must not be overlooked. The above-mentioned points are the main reasons why its high theoretical enthalpy of combustion cannot be achieved in practical applications.

In order to achieve complete combustion before being exhausted, the reactivity of Al particles is becoming an important area of research toward advancing energetic material science from both a processing as well as a combustion perspective. It is well known that the chemical and physical properties of nano-sized particles can differ substantially from those observed in micro-sized particles. Nano-sized Al particles are more reactive than micro-sized Al (μAl) particles toward oxidation and other reactions as a result of their higher specific surface areas [12]. However, the thickness of the Al_2_O_3_ shell is independent of the particle size, and the combustion mechanism of Al particles with different particle sizes is still a matter of debate [13,14,15,16,17]. According to the current research situation, the diffusion oxidation mechanism (DOM) [18] of Al NPs at a slow heating rate is widely accepted to describe its combustion process, involving the collision of oxygen with the Al NPs and then the subsequent transport through oxidation products.

In fact, fluorine is the most electronegative element known, and the Al-F bond (664 ± 6 kJ·mol^−^^1^) is stronger than the Al-O bond (512 ± 4 kJ·mol^−^^1^) [19]. Undoubtedly, the exothermic value of a fluorination reaction (1510 kJ·mol^−^^1^) is much higher than that of an oxidation reaction (839.4 kJ·mol^−^^1^) on a molar basis for the oxidation product [20]. Thence, the science of Al NPs’ reactivity with fluorine-containing oxidizers has important implications for energy-generating materials. Typically, the Al_2_O_3_ shell is inert in the combustion process of Al NPs. Interestingly, fluorine atoms could decompose the inert Al_2_O_3_ shell surrounding the Al NPs into AlF_3_ products, producing an exothermic surface reaction that promotes the decomposition of the fluorine-containing oxidizers [21,22]. It has been reported that fluorine-containing oxidizers such as polytetrafluoroethylene (PTFE) [23,24], polyvinylidene fluoride (PVDF) [25,26,27], perfluorotetradecanoic acid (PFTD) [28,29], perfluoroalkyl acids (PFAA) [30], and perfluorohexadecanoic acid (PFHD) [31] are capable of enhancing the ignition and combustion behavior of Al NPs because of pre-ignition reaction (PIR) which occurs prior to the oxidation reaction of the Al core. However, although the effect of PIR on activating reactivity of Al NPs was demonstrated several years ago [32,33], little attention has been paid to the oxidation mechanism of surface-modification Al NPs regulated by the reaction interface Fuel-Oxidizer ratio.

Compared with traditional fluorine-containing oxidizers, perfluoropolyether (PFPE) has attracted interest from several different fields because of its unique combination of advantageous physical and chemical properties (i.e., it has an excellent thermal and oxidation stability, enabling it to be used in applications requiring a wider temperature range) [34,35]. Nonetheless, the use of PFPE in micro-sized/nano-sized Al particles has rarely been reported thus far [19,36,37]. This is due to the lack of a suitable carrier fluid for PFPE, which would lead to the evident agglomeration of Al particles. Recently, McCollum et al. [38] investigated the reaction kinetics and combustion performance of thermite (Al/MoO_3_ or Al/CuO) blended with varying percentages of PFPE. It is rather remarkable that thermite is the research object, and the bond dissociation energy of the metal oxide plays an indispensable part in improving the overall reactivity of Al NPs.

With the above-mentioned views, the objective of this work is to construct PFPE-functionalized Al NPs using a facile fabrication method to determine how their oxidation mechanism is regulated by the reaction interface Fuel-Oxidizer ratio. The PFPE-functionalized Al NPs were obtained and fundamentally characterized in terms of their microstructure. The oxidation mechanism and constant volume combustion characteristics of the PFPE-functionalized Al NPs were also investigated by thermal analysis and combustion performance evaluation experiments.

## 2. Experimental Section

### 2.1. Materials

Nano aluminum (nAl) with an average diameter of 150 nm was supplied by Hefei AVIC Nano Technology (Hefei, China) Development Co., Ltd. The Al NPs were covered with Al_2_O_3_ with a thickness of about 2.5~3.0 nm. The Fomblin^®^ Y 25 PFPE produced by SOLVAY (Brussels, Belgium) was used as the fluorine-containing oxidizer to modify Al NPs. 1,1,2-trichloro-1,2,2-trifluoroethane was purchased from Sigma-Aldrich (Shanghai, China) Trading Co., Ltd., and used as a carrier fluid to disperse PFPE.

### 2.2. Preparations

**Figure 1:**
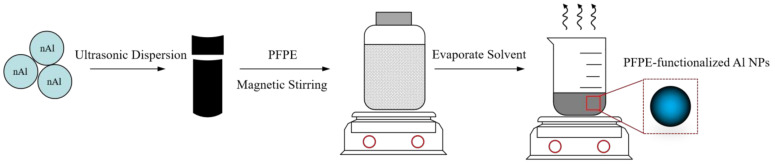
Schematic description of the fabrication of PFPE-functionalized Al NPs.

Al NPs and the PFPE were weighed with a predetermined ratio (Table S1) and suspended in 1,1,2-trichloro-1,2,2-trifluoroethane. The solution was then mixed via a magnetic stirrer at 600 rpm for 45 min and poured into a beaker. The 1,1,2-trichloro-1,2,2-trifluoroethane was evaporated in a fume hood until the remaining mass was only that of PFPE-functionalized Al NPs (e.g., about 24 h). Finally, PFPE-functionalized Al NPs were obtained for further measurements. The schematic description of PFPE-functionalized Al NPs is shown in Figure 1.

### 2.3. Characterizations

Field emission transmission electron microscopy (FE-TEM, Tecnai G2 F30, FEI, Hillsboro, OR, USA) was used to examine the micro-structure of the PFPE-functionalized Al NPs. Furthermore, after ultrasonic dispersion for 30 min, the particle size distribution was analyzed by a laser particle size analyzer (Mastersizer 2000, Malvern Panalytical, Malvern, UK). The element distribution of the PFPE-functionalized Al NPs was scanned by an energy dispersive spectrometer (EDS) equipped in the TEM device. X-ray photoelectron spectroscopy (XPS, Escalab 250Xi, Thermo, Waltham, MA, USA) was performed to analyze the chemical state of elements. Thermogravimetric analysis (TGA) and differential scanning calorimetry (DSC, STA449F3, NETZSCH, Selb, Germany) were simultaneously conducted at a heating rate of 5 K·min^−1^ from room temperature to 1473 K in air atmosphere to study the thermal reaction behaviors of the PFPE-functionalized Al NPs. The phases of the PFPE-functionalized Al NPs before and after oxidation were analyzed with X-ray diffraction (XRD, D8 ADVANCE, BRUCKER, Karlsruhe, Germany) using Cu Kα radiation (λ = 1.54180 Å), and operating under the condition of 40 kV/40 mA. The 2θ range measured was 5–90° with steps of 0.02°/0.1 s. A scanning electron microscope (SEM, SU8020, Hitachi, Tokyo, Japan) was used to observe the morphology of the oxidation products. The content of active aluminum of the samples was measured by a testing device (as shown in Figure S1) based on the volume of hydrogen generated by means of reacting with a specific concentration of hydrochloric acid.

### 2.4. Calorific Value Measurement

A microcomputer automatic calorimeter (MAC, TRHW-7000C, Hebi Tianrun Electronic Technology Co., Ltd., Hebi, China) was used to determine the calorific value of PFPE-functionalized Al NPs. In this experiment, approximately 0.3 g of the samples were put into the calorimeter inflated with oxygen under 3 MPa, and the heat release was measured.

### 2.5. Constant Volume Combustion Cell Test

A constant volume combustion cell test (as shown in Figure S2) of 30 mg samples was conducted by the flame ignition method with an oxygen atmosphere (~2 MPa) to characterize the energy response and work capacity of the PFPE-functionalized Al NPs. The flame was generated by the tip of a nichrome wire, which was heated by a controlled DC current. The change in the pressure generated by the vigorous oxidation process was recorded by the sensor as a function of time.

## 3. Results and Discussion

### 3.1. Micro-Structure Analysis

**Figure 2:**
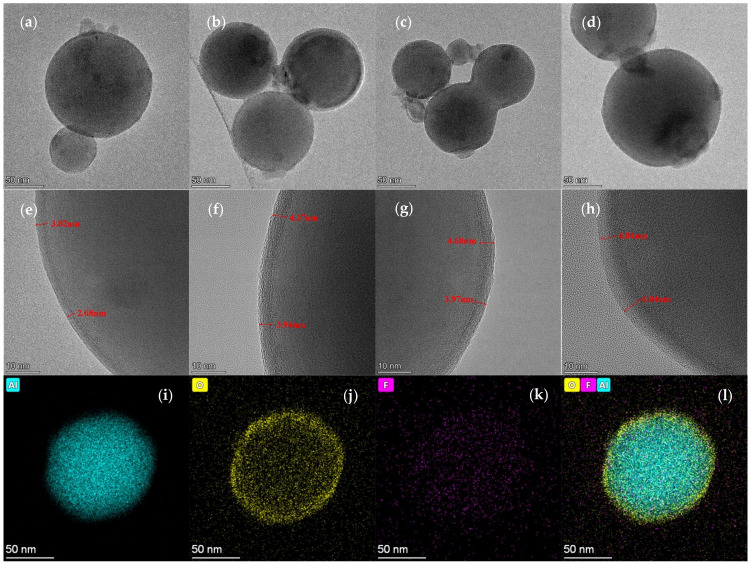
TEM photos of (**a**) Al NPs, (**b**) nAl@2.5%PFPE particles, (**c**) nAl@5.0%PFPE particles, (**d**) nAl@5.0%PFPE particles; high-resolution TEM photos of (**e**) Al NPs, (**f**) nAl@2.5%PFPE particles, (**g**) nAl@5.0%PFPE particles, (**h**) nAl@5.0%PFPE particles; and EDS results showing the distributions of (**i**) Al element (green), (**j**) O element (yellow), (**k**) F element (purple), and (**l**) above-mentioned elements.

TEM photos were captured to characterize the micro-structural differences between the Al NPs and PFPE-functionalized Al NPs. As shown in Figure 2a–d, the morphology of the Al NPs and PFPE-functionalized Al NPs is approximately spherical with a typical core–shell structure. In addition, it can be seen from Figure 2e that the surface of the Al NPs has an extremely obvious amorphous oxide layer with a thickness between 2.68 nm and 3.82 nm. In contrast, the amorphous layer thickness (δ) on the surface of the PFPE-functionalized Al NPs is significantly greater than the surface oxide layer of Al NPs. In the example illustration, the δ_min_ of the nAl@2.5%PFPE particles is 3.94 nm (Figure 2f). Moreover, the δ_min_ of the nAl@5.0%PFPE particles is 3.97 nm (Figure 2g), which is even thicker than the surface oxide layer of Al NPs. Moreover, the amorphous layer is tightly adsorbed on the surface of the Al NPs, and there are no exposed spherical Al NPs. As the mass fraction of PFPE increases, the thickness of the amorphous layer correspondingly increases. The δ_max_ of the nAl@7.5%PFPE particles even reached 6.04 nm (Figure 2h).

To confirm whether PFPE is distributed on the surface of Al NPs, Al, O, and F elements of samples were scanned by an EDS equipped in the TEM device. The element distribution of the individual nAl@2.5%PFPE particles is shown in Figure 2i–l; corresponding characterization results of the individual nAl@5.0%PFPE and nAl@7.5%PFPE particle are shown in Figure S3 and Figure S4, respectively.

A clear contrast is observed in high-angle annular dark-field (HAADF) TEM images, further confirming the core–shell structure of Al NPs. Al element is homogeneously dispersed throughout the particle. O element is widely distributed at the boundary because of the presence of the Al_2_O_3_ shell. The uniform distribution of F element on the surface of the spherical particle further proves that PFPE has been evenly surrounded on the surfaces of the Al NPs.

**Figure 3:**
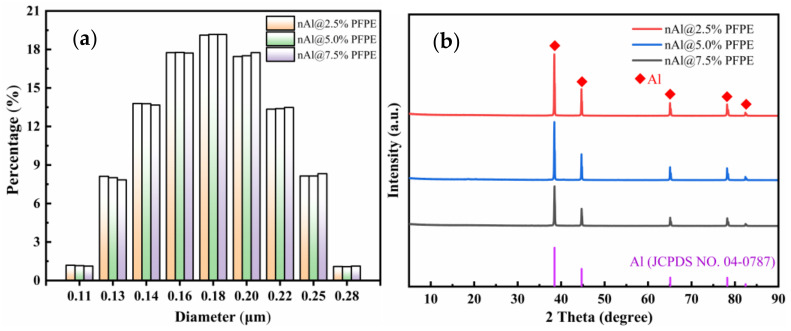
Particle size distribution curve (**a**) and X-ray diffraction patterns (**b**) of PFPE-functionalized Al NPs.

In order to observe the particle size distribution of PFPE-functionalized Al NPs in detail, the particle size distribution was also characterized by a laser particle size analyzer, as shown in Figure 3a. The particle size distribution curve illustrates PFPE-functionalized Al NPs basically have a normal distribution with no obvious agglomeration. Compared with the original Al NPs (D_50_: ~150 nm), the average particle size of PFPE-functionalized Al NPs is larger than that of original Al NPs. No occurrence of other abnormal states indicates the feasibility of the preparation method. Moreover, with the increase in the PFPE content in the Al NPs, the particle size of the PFPE-functionalized Al NPs does not change significantly, but it increases slightly.

The XRD patterns shown in Figure 3b confirm that the crystal structure of Al NPs does not change after surface modification. In agreement with the TEM analysis, this result also indicates that the composite assembly process of PFPE-functionalized Al NPs is physical mixing, with no new material appearing.

### 3.2. X-ray Photoelectron Spectroscopy Analysis

**Figure 4:**
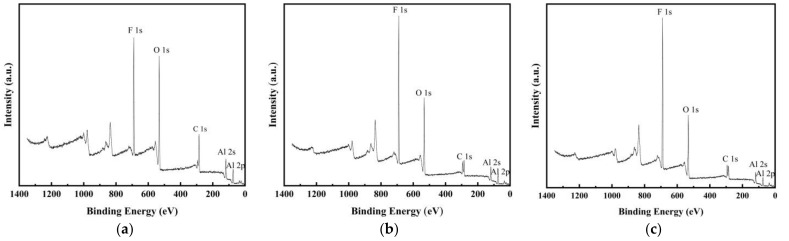
XPS spectra for (**a**) nAl@2.5%PFPE particles, (**b**) nAl@5.0%PFPE particles, and (**c**) nAl@7.5%PFPE particles.

To identify the presence of PFPE on the surface of Al NPs, XPS was performed on PFPE-functionalized Al NPs, as shown in Figure 4. As expected, C 1s peaks, F 1s peaks, O 1s peaks, Al 2s, and Al 2p peaks were observed in PFPE-functionalized Al NPs, revealing the interfacial contact between Al NPs and PFPE.

**Figure 5:**
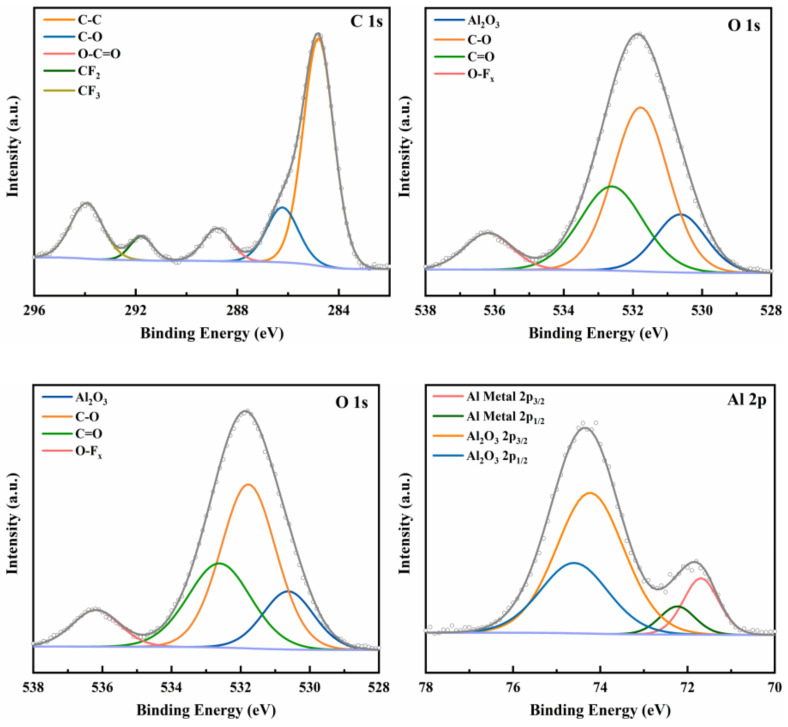
XPS spectra of C 1s peaks, O 1s peaks, F 1s peaks, and Al 2p peaks for nAl@2.5%PFPE particles.

To determine the adsorption form of PFPE in PFPE-functionalized Al NPs, the chemical states of C, F, O, and Al elements were analyzed by means of peak-differentiating and imitating, as shown in Figure 5 (Figures S5 and S6). Upon closer inspection, the XPS spectra of C 1s peaks in nAl@2.5%PFPE particles show the presence of C-C (284.8 eV), C-O (286.2 eV), O-C=O (288.8 eV), CF_2_ (291.8 eV), and CF_3_ (293.9 eV), all of which arise from PFPE. The deconvolution of the O 1s signal shows the presence of Al_2_O_3_ (530.6 eV), C-O (531.8 eV), C=O (532.6 eV), and O-F_x_ (536.2 eV). This result is in good agreement with the physical structure of PFPE-functionalized Al NPs. The F 1s spectra have only one peak corresponding to the C-F bond (689.6 eV). Moreover, the peaks at 74.2 eV and 74.6 eV are metallic Al 2p1/2 and 2p3/2, respectively. The peaks at 71.7 eV and 72.2 eV are indicative of Al-O bonds. The above results indicate that there is no bonding between Al and F. On the other hand, it can be speculated that PFPE is coated on the surface of Al NPs by physical adsorption due to the low surface tension of PFPE.

### 3.3. Oxidation Mechanism Analysis

**Figure 6:**
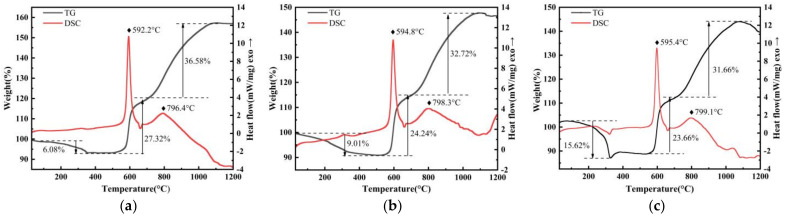
TG-DSC curves of (**a**) nAl@2.5%PFPE particles, (**b**) nAl@5.0%PFPE particles, (**c**) nAl@7.5%PFPE particles.

Thermogravimetric analysis and a differential scanning calorimetry analysis were conducted to investigate the oxidation mechanism of PFPE-functionalized Al NPs in air atmosphere at a heating rate of 5 K·min^−1^ from room temperature to 1473 K, as shown in Figure 6. In order to further understand the oxidation mechanism, the reaction process can be divided into three stages including condensed-phase reaction, initial oxidation, and final oxidation. The characteristic parameters are shown in Table S2, where Δ*m*_1_, Δ*m*_2_, and Δ*m*_3_ are the mass percentages and *T_p_*_1_, *T_p_*_2_, and *T_p_*_3_ are the peak temperatures of each stage.

The PFPE-functionalized Al NPs lose weight in the first stage, and the weight loss was basically positively correlated with the mass fraction of the fluorine-containing oxidizer PFPE. There is clearly a small exothermic change at the temperature range of 320–400 °C shown in the DSC curves of PFPE-functionalized Al NPs, especially in the heat flow curves of nAl@7.5%PFPE particles, which is due to the fact that its decomposition products decompose the alumina shell surrounding the Al NPs into AlF_3_ products (pre-ignition reaction) before oxidation reaction of the Al NPs, as shown in Equation (1).

Al_(surface)_ + 3F_(PFPE)_ → AlF_3_ + 1510 kJ·mol^−1^ (1)

It is noted that reaction interface Fuel-Oxidizer ratio (*Φ*) regulates the weight loss in the first stage of nAl, as shown in Equation (2), where *m* is the mass, the fuel is nAl, and the oxidizer is PFPE. The coefficient *Φ* also indicates the ratio of surface oxide to fluorine-containing oxidizer PFPE when the mass of the Al remains constant.

(2) $$\Phi ={\left[\frac{m_{fuel}}{m_{oxidizer}}\right]}_{actual}$$

In addition, the condensed-phase reaction describes the participation of PFPE in the oxidation reaction of Al NPs, as shown in Equation (3), where *a*, *b*, *c*, and *d*- are coefficients used to balance the oxidation reaction and *P* represents other products of the reaction.

(3) $$a{\mathrm{CF}}_{3}O-{\left[-\mathrm{CF}\left({\mathrm{CF}}_{3}\right){\mathrm{CF}}_{2}O-\right]}_{x}{\left(-{\mathrm{CF}}_{2}O-\right)}_{y}-{\mathrm{CF}}_{3}+b\mathrm{Al}\to c{\mathrm{Al}}_{2}O_{3}+d{\mathrm{AlF}}_{3}+P$$

In the second stage, the melting point of Al has not yet been reached. The weight gain of nAl@2.5%PFPE particles (Figure 6a), nAl@5.0%PFPE particles (Figure 6b), and nAl@7.5%PFPE particles (Figure 6c) at this stage is 27.32%, 24.24%, and 23.66%, respectively. It is noted that the oxidation reaction at this stage is independent of coefficient *Φ*. However, it cannot be ignored in the interface reaction of this type of MICs. The weight gain is due to the formation of feasible diffusion paths caused by the pre-ignition reaction between PFPE and surface oxide in the first stage. Here, we also provide the physical properties of Al and Al_2_O_3_ [39] to analyze the thermal reaction behaviors of PFPE-functionalized Al NPs, as shown in Table S3. Al (23 × 10^−6^) has a larger thermal expansion coefficient than Al_2_O_3_ (8.6 × 10^−6^), that is, the ratio of thermal expansion coefficient of Al to Al_2_O_3_ is greater than 1. The Al_2_O_3_ shell is in a tensile state, while the inner Al is in a compressed state, and the internal stress increases with the increase in temperature. This phenomenon leads to an increase in diffusive oxygen exchange in diffusive boundary layers, and the weight gain of PFPE-functionalized Al NPs increases sharply at this stage.

In the third stage, the Al core has melted and its volume expands by 12.5% (ρ_Al(l)_ = 2380 kg·m^−3^), ignoring the thermal expansion of the Al_2_O_3_ shell. Under such extreme conditions, the shell will not be able to withstand the internal pressure, causing the shell to rupture. The exposed surface of Al NPs is rapidly oxidized at a high temperature, which speeds up the growth of γ-Al_2_O_3_, deepens the conversion degree, and increases the heat release. Meanwhile, with the crystalline phase transition of γ-Al_2_O_3_ → δ-Al_2_O_3_ → θ-Al_2_O_3_ → α-Al_2_O_3_ [40], the oxide layer gradually thickens to reach the Al core.

**Figure 7:**
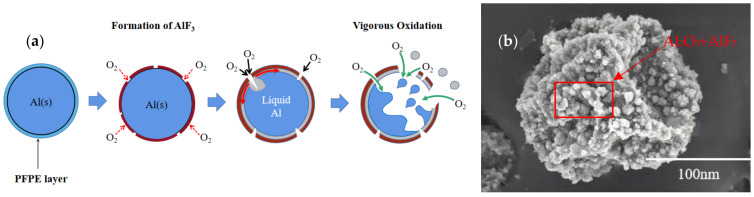
(**a**) Schematic description of oxidation mechanism of PFPE-functionalized Al NPs and (**b**) enlarged SEM image of the oxidized PFPE-functionalized Al NPs.

The weight gain of Al NPs with varying percentages of PFPE during the entire oxidation process is more than that of Al NPs with a median particle diameter of 200 nm reported in the literature [11]. Thus, adding a certain amount of fluorine-containing oxidizer PFPE can deepen the oxidation depth of Al NPs, but there is no linear correlation between them. In conclusion, ignoring the influence of a small amount of impurities, the oxidation mechanism of PFPE-functionalized Al NPs can be seen in Figure 7. In particular, the surface fluorination reaction mechanism plays an indispensable part in the initial stage of oxidation; as a first step, elementary exchange reactions in which OH- is replaced with F- are reported in the literature [33], as shown in Equation (4). In this reaction, the Al-F bond forms after fluorine dislodges hydroxyls from the Al_2_O_3_ passivation surface.

X-F + Al_2_O_3_-OH → Al_2_O_3_-F + X-OH (4)

### 3.4. The Calorific Value Analysis

In order to evaluate the applicability of PFPE-functionalized Al NPs in the energetic system, the effect of the content of active aluminum on the calorific value of PFPE-functionalized Al NPs was studied in this work, compared with nAl.

The results show that the content of active aluminum: nAl (85.84 wt%) ≥ nAl@2.5%PFPE (85.61 wt%) > nAl@5.0%PFPE (82.93 wt%) > nAl@7.5%PFPE (80.86 wt%). This result provides strong evidence that PFPE prevents the further oxidation of Al NPs under natural conditions. Due to its high thermal and oxidative stability, PFPE can effectively block the contact between Al NPs and air. Simultaneously, as the PFPE content increases, the content of active aluminum of the PFPE-functionalized Al NPs will correspondingly decrease. The reason is that the percentage of amorphous layer gradually increases. This phenomenon is consistent with the results of Section 3.1. However, compared with micro-sized Al particles, Al NPs have a larger specific surface area and higher reactivity, which results in the oxide layer percentage of nAl being higher than that of μAl under natural conditions. That is the reason why the content of active aluminum of nAl is not as good as that of μAl as reported in the literature.

As shown in Table 1, after adding different contents of PFPE, the calorific value of the modified Al NPs is increased by 7.22%, 4.80%, and 3.21%, respectively. Although the calorific value of PFPE is lower than Al, the influence of PFPE on the energy release of Al NPs cannot be ignored. Due to the participation of surface fluorination of Al NPs, the energy efficiency of PFPE-functionalized Al NPs has improved compared with Al NPs. When it comes to the reason for the above phenomenon, mainly the surface exothermic fluorination between the inert Al_2_O_3_ shell and fluorine would excite the overall reactivity of Al NPs, which is regulated by the reaction interface Fuel-Oxidizer ratio.

**Figure 8:**
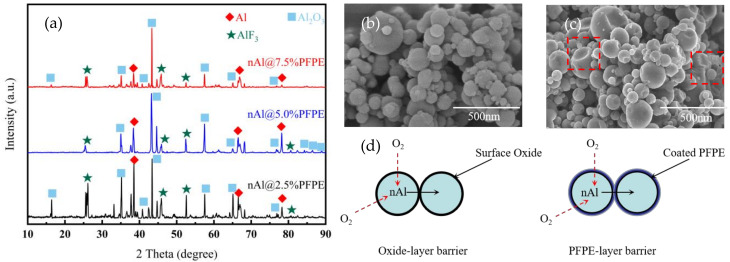
(**a**) X-ray diffraction patterns of the oxidized PFPE-functionalized Al NPs; SEM photos of (**b**) the initial and (**c**) the oxidized PFPE-functionalized Al NPs; (**d**) oxidation paths of PFPE-functionalized Al NPs compared with nAl.

Here, we provide a comparison of the XRD patterns after the oxidation process, as shown in Figure 8a. This result demonstrates that the oxidized PFPE-functionalized Al NPs still contain a small amount of Al because of incomplete reactions. In addition, the combustion products Al_2_O_3_ and AlF_3_ are observed from the XRD patterns. During the combustion process, the Al core erupts and overflows due to phase transformation. It is speculated that Al_2_O_3_ is caused by the combustion of nAl, and AlF_3_ is generated due to the PIR that occurs after PFPE is decomposed, which causes the free fluorine-containing fragments to combine with the Al_2_O_3_ shell by substituting hydroxyl. Moreover, it can be seen from Figure 8b,c the morphology differences of the initial and the oxidized PFPE-functionalized Al NPs are captured, and the rupture of Al NPs is in good agreement with the schematic description of the oxidation mechanism of PFPE-functionalized Al NPs. Interestingly, the exposed surface after the sublimation of AlF_3_ (Sublimation temperature: 1276 °C) [41] can provide efficient oxidation paths where the inner Al rapidly encounters external oxygen atoms without hindering the oxidation of nAl (Figure 8d), thereby increasing the exothermic reaction rate and the exothermic reaction energy.

### 3.5. Constant Volume Combustion Characteristics

**Figure 9:**
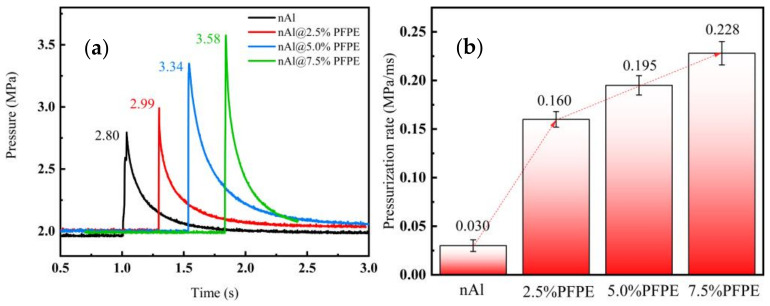
Comparison of (**a**) the pressure changes with time and (**b**) the pressurization rate of nAl and PFPE-functionalized Al NPs.

In order to characterize the energy response and work capacity of PFPE-functionalized Al NPs, the pressure changes with time were measured by the ignition of PFPE-functionalized Al NPs in a closed chamber. As shown in Figure 9a, higher peak pressure could be achieved with higher content of PFPE and is higher than that of nAl. In this work, we used the velocity of the change in the pressure to describe the vigorous oxidation process, which can be quantitatively expressed as the pressurization rate (MPa/ms), as shown in Equation (5).

*Pressurization rate* = (*P_max_* − *P_i_*)/(*t_max_* − *t_i_*) (5)

Here, *P_max_* is the maximum pressure, *P_i_* is the pressure when the ignition process begins, *t_max_* is the time until the pressure reaches its maximum value, and *t_i_* is the ignition time.

Figure 9b clearly shows the improvement in the pressurization rate of the PFPE-functionalized Al NPs. The pressurization rate of PFPE-functionalized Al NPs is much higher than that of nAl (0.030 MPa/ms), which is due to the rapid heat release and gaseous phases such as CO_2_ and HF generated by the thermal decomposition of PFPE. Interestingly, similar results were found from the data on energy efficiency.

## 4. Conclusions

The PFPE-functionalized Al NPs with tunable reactivity were designed and fabricated by using a facile fabrication method. The prepared novel core–shell PFPE-functionalized Al NPs had a different level of reactivity depending on the thickness of the PFPE layer. The following conclusions were obtained:

(1) PFPE-functionalized Al NPs with varying percentages of PFPE were constructed using the solvent suspension method. The micro-structure of the PFPE-functionalized Al NPs basically had a normal distribution with no obvious agglomeration. PFPE dispersed homogeneously throughout Al NPs by means of physical adsorption.
(2) The oxidation mechanism of PFPE-functionalized Al NPs at a slow heating rate was regulated by the reaction interface Fuel-Oxidizer ratio. Under the effect of an exothermic surface reaction between the PFPE and Al_2_O_3_ shell, the active aluminum contained in the aluminum core was more likely to participate in the vigorous oxidation reaction.
(3) After adding different contents of PFPE, the calorific value of the modified Al NPs was increased by 7.22%, 4.80%, and 3.21%, respectively. Simultaneously, compared with Al NPs, the exothermic reaction rate and the exothermic reaction energy were all increased because of the decomposition products participating in the interface reaction.

## Figures and Tables

**Table 1:** Test results of the calorific value and calculated energy efficiency.

Samples	Calorific Value (MJ·kg^−1^)	Energy Efficiency (%)
nAl	23.95	89.86
nAl@2.5%PFPE	25.68	96.60
nAl@5.0%PFPE	25.10	97.48
nAl@7.5%PFPE	24.72	98.46

## Data Availability

Data are contained within the article or supplementary material. The data presented in this study are available in the Supplementary Material.

## Supplementary Materials

The following supporting information can be downloaded at: https://www.mdpi.com/article/10.3390/nano12030530/s1: Figure S1: Testing device for the content of active aluminum: (1) thermometer; (2) eudiometer; (3) weighing bottle; (4) water tank; (5) gas vent; (6) piston; (7) level bottle; (8) conical flask. Figure S2: Schematic description of the constant volume combustion cell test: (1) constant volume combustion cell; (2) pressure testing device; (3) controlled DC current; (4) data acquisition unit. Figure S3: The elements distribution of individual nAl@5.0%PFPE particle: (a) Al element (green), (b) O element (yellow), (c) F element (purple), (d) above-mentioned elements. Figure S4: The elements distribution of individual nAl@7.5%PFPE particle: (a) Al element (green), (b) O element (yellow), (c) F element (purple), (d) above-mentioned elements. Figure S5: XPS spectra of C 1s peaks, O 1s peaks, O 1s peaks and Al 2p peaks for nAl@5.0%PFPE particles. Figure S6: XPS spectra of C 1s peaks, O 1s peaks, O 1s peaks and Al 2p peaks for nAl@7.5%PFPE particles. Table S1: Weight percent of all components in each sample. Table S2: Characteristic parameters of PFPE-functionalized Al NPs at a heating rate of 5 K·min^−^^1^. Table S3: Physical properties of Al and Al_2_O_3_.
