# Supplementary material for: Tuning the Reactivity of Perfluoropolyether-Functionalized Aluminum Nanoparticles by the Reaction Interface Fuel-Oxidizer Ratio
Source: Nanomaterials (Basel). 2022 Feb 3;12(3):530. doi: 10.3390/nano12030530 (PMC8838337; doi:10.3390/nano12030530)
Supplement: Supplementary file 1 [file nanomaterials-12-00530-s001.zip › nanomaterials-1567262-supplementary.pdf]

nanomaterials

Supplementary Materials

Tuning the Reactivity of Perfluoropolyether-Functionalized Aluminum Nanoparticles by the Reaction Interface Fuel-Oxidizer Ratio

Chengcheng Wu [1], Jianxin Nie [1], Shengwei Li [1], Wei Wang [1,2], Qi Pan [1], and Xueyong Guo [1,*]

1 State Key Laboratory of Explosion Science and Technology, Beijing Institute of Technology, Beijing 100081, China; 3120185179@bit.edu.cn (C.W.); niejx@bit.edu.cn (J.N.); 3120210240@bit.edu.cn (S.L.); 3220185029@bit.edu.cn (W.W.); panqi97@163.com (Q.P.)
2 Beijing Institute of Space Long March Vehicle, China Academy of Launch Vehicle Technology, Beijing 100074, China
* Correspondence: nust@bit.edu.cn

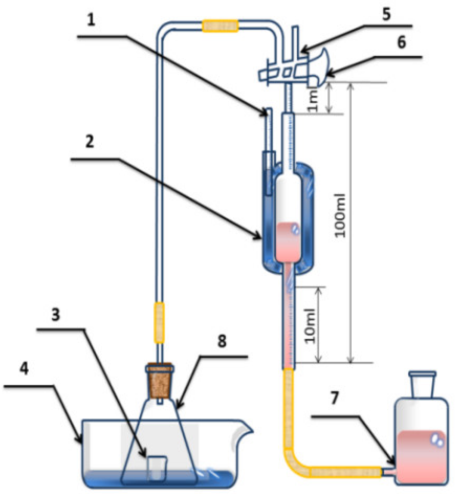

Figure S1. Testing device for the content of active aluminum: 1) thermometer; 2) eudiometer; 3) weighing bottle; 4) water tank; 5) gas vent; 6) piston; 7) level bottle; 8) conical flask.

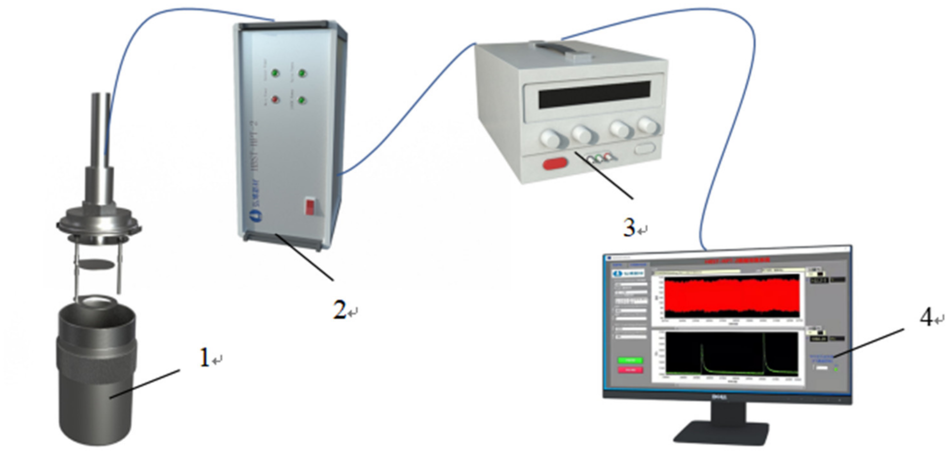

Figure S2. Schematic description of the constant volume combustion cell test: 1) constant volume combustion cell; 2) pressure testing device; 3) controlled DC current; 4) data acquisition unit.

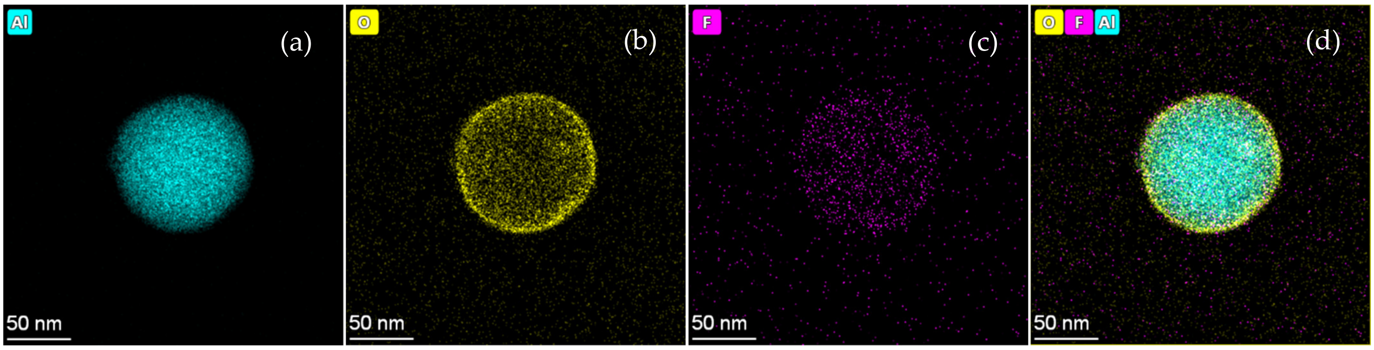

Figure S3. The elements distribution of individual nAl@5.0%PFPE particle: (a) Al element (green), (b) O element (yellow), (c) F element (purple), (d) above-mentioned elements.

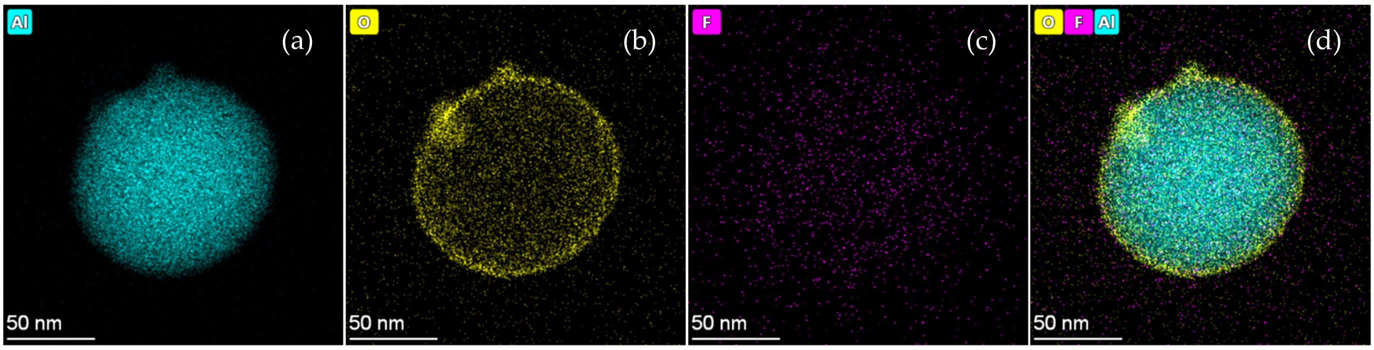

Figure S4. The elements distribution of individual nAl@5.0%PFPE particle: (a) Al element (green), (b) O element (yellow), (c) F element (purple), (d) above-mentioned elements.

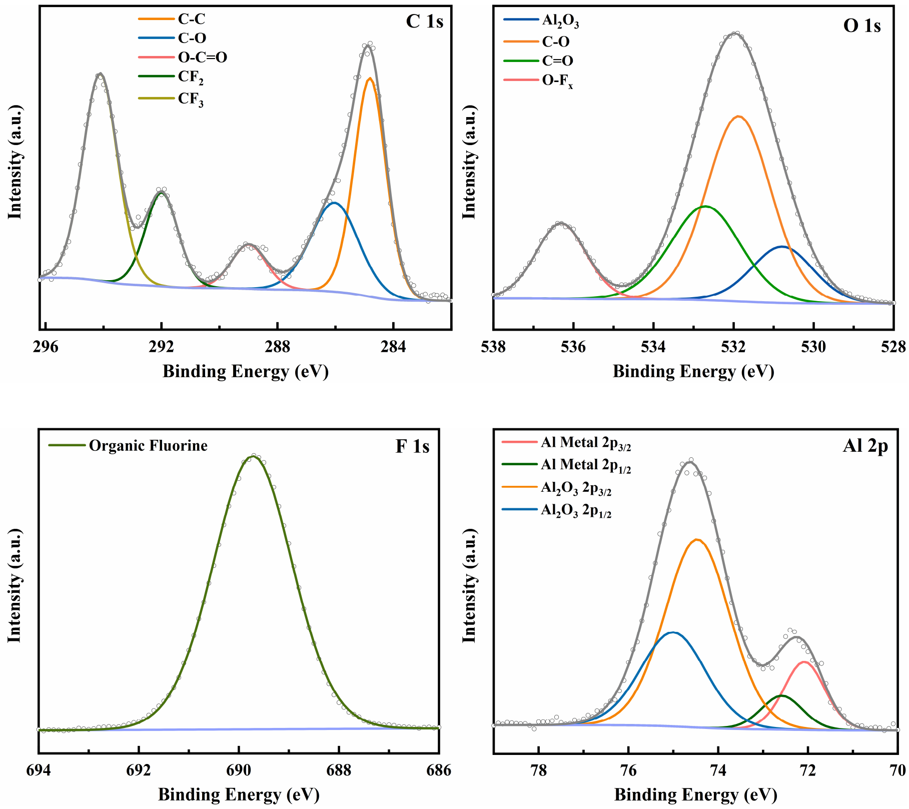

Figure S5. XPS spectra of C 1s peaks, O 1s peaks, O 1s peaks and Al 2p peaks for nAl@5.0%PFPE particles.

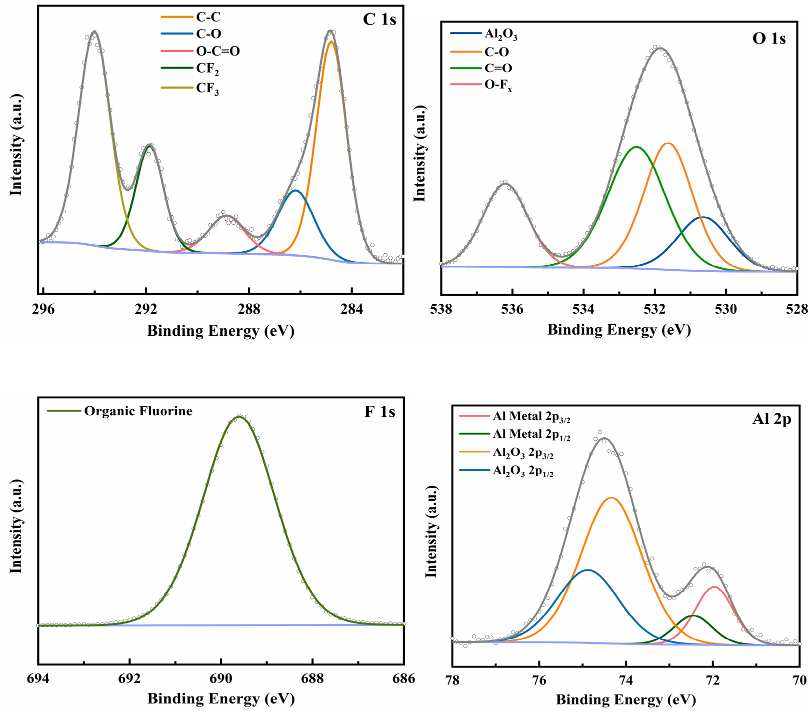

Figure S6. XPS spectra of C 1s peaks, O 1s peaks, O 1s peaks and Al 2p peaks for nAl@7.5%PFPE particles

Table S1. Weight percent of all components in each sample.

Samples	Al NPs (%)	PFPE (%)	1,1,2-trichloro-1,2,2-trifluoroethane (CFC-113)
nAl@2.5%PFPE	97.5	2.5	1g Al NPs and PFPE suspended in 60 mL of CFC-113
nAl@5.0%PFPE	95.0	5.0	
nAl@7.5%PFPE	92.5	7.5	

Table S2. Characteristic parameters of PFPE-functionalized Al NPs at a heating rate of 5K·min^{-1}.

Samples	Δm_1 (%)	Δm_2 (%)	Δm_3 (%)	T_{p1} (℃)	T_{p2} (℃)	T_{p3} (℃)
nAl@2.5%PFPE	-6.08	27.32	36.58	316.3	592.2	796.4
nAl@5.0%PFPE	-9.01	24.24	32.72	319.3	594.8	798.3
nAl@7.5%PFPE	-15.62	23.66	31.66	322.6	595.4	799.1

Table S3. Physical properties of Al and Al_2O_3

Physical properties	Al	Al_2O_3
Molar mass (g·mol^{-1})	26.98	101.96
Density (kg·m^{-3})	2700	4000
Melting point (K)	933	2327
Boiling point (K)	2740	3373
Specific heat capacity (J·kg^{-1}·K^{-1})	938	880
Thermal conductivity (W·m^{-1}·K^{-1})	210	18
Thermal expansion coefficient (K^{-1})	23×10^{-6}	8.6×10^{-6}
Shear modulus (GPa)	26	124
Elastic modulus (GPa)	70~79	300
Bulk modulus (GPa)	76	165
